# Mental health by native–immigrant intermarriage in Sweden: a register-based retrospective cohort study

**DOI:** 10.1093/eurpub/ckac158

**Published:** 2022-11-15

**Authors:** Helena Honkaniemi, Sol Pía Juárez, Mikael Rostila

**Affiliations:** Department of Public Health Sciences, Stockholm University, Stockholm, Sweden; Centre for Health Equity Studies (CHESS), Stockholm University/Karolinska Institutet, Stockholm, Sweden; Department of Public Health Sciences, Stockholm University, Stockholm, Sweden; Centre for Health Equity Studies (CHESS), Stockholm University/Karolinska Institutet, Stockholm, Sweden; Department of Public Health Sciences, Stockholm University, Stockholm, Sweden; Centre for Health Equity Studies (CHESS), Stockholm University/Karolinska Institutet, Stockholm, Sweden

## Abstract

**Background:**

Native–immigrant intermarriage is often regarded as a proxy for integration, given that intermarried immigrants are more socioeconomically and culturally similar to natives than intramarried immigrants. This study aimed to assess whether integration affects mental health and care-seeking behaviours, examining prescription hazards for psychotropic medications by native–immigrant marital composition in Sweden.

**Methods:**

Total population register data were used to identify first-time married couples residing in Sweden between 31 December 2005 and 31 December 2016. Index persons were distinguished by gender and partners’ origin (native vs. immigrant), as well as by immigrants’ regions of origin, with intramarried natives as references. Using Cox regression, hazard ratios (HRs) with 95% confidence intervals (95% CIs) were calculated for antidepressant and anxiolytic prescriptions and adjusted for socioeconomic factors, presence of children and length and quality of marriage.

**Results:**

Intramarried immigrant women had higher psychotropic prescription hazards than intramarried native references (HR 1.11, 95% CI 1.10–1.12), whereas intermarried immigrant women had equal hazards. Immigrant women’s hazards were lower than native references after adjustment. Intramarried immigrant men had the greatest prescription hazards (HR 1.33, 95% CI 1.32–1.34), and intermarried immigrant men slightly higher hazards (HR 1.11, 95% CI 1.10–1.13), than intramarried natives. All were partly attenuated after adjustment. Intermarriage hazards increased by similarities in regions of origin, especially among men.

**Conclusions:**

Integration indicated by intermarriage appears to be protective for the mental health of immigrants, especially for immigrant men. Future research should empirically disentangle the social, cultural and socioeconomic mechanisms underlying these health differences.

## Introduction

Immigrants have generally been shown to have worse mental health than natives, both internationally^1^ and specifically in Sweden.^2^ However, measures of psychiatric care use, including prescriptions of psychotropic medications, reveal mixed findings. Psychotropic prescription risks vary widely by immigrants’ gender, region of origin and duration of residence in the host country, across immigrant groups and compared to natives in Sweden^3–6^ and other European countries.^7–9^ However, evidence on the influence of immigrant integration on mental health, including psychotropic prescription receipt, is limited.

The ‘ultimate litmus test’ of immigrant integration, intermarriage,^10^ represents an end stage of the ‘two-way process of mutual adaptation’ between immigrants and natives.^11^ Immigrants married to natives have been found to have better socioeconomic attainment than their intramarried counterparts (i.e. those married to other immigrants), whether through selection into intermarriage or socioeconomic gains during marriage.^12–14^ Furthermore, intermarriage can indicate or foster the acquisition of native norms and behaviours, access to native social networks and feelings of belonging in a new society,^15^^,^^16^ while being associated with fewer healthcare-specific obstacles, including language barriers and lack of institutional awareness.^17^ Together, this suggests that integration through intermarriage could either correspond to decreased levels of psychotropic prescription receipt through improved social circumstances,^18^ or increased levels of receipt by reducing barriers to healthcare uptake.^19–21^

Intermarriage has been studied in relation to a number of health outcomes,^17^^,^^22^^,^^23^ including mental health, with evidence of both higher and lower risks of self-reported depression and suicide for intermarried than intramarried immigrants in Sweden and Europe.^24^^,^^25^ However, evidence of more comprehensive measures of mental health, including psychotropic prescriptions, is lacking. This study will investigate differences in prescriptions of psychotropic medications by married couples’ nativity and immigrants’ regions of origin in Sweden, for both men and women. The analyses will utilize total population register data with comprehensive information on psychotropic prescriptions and potential confounders in the form of socioeconomic, familial and marital factors. Its findings will help to highlight the mental health consequences of complex, co-occurring integration processes across heterogenous immigrant groups, with potential implications for reducing health inequalities among immigrants and between natives and immigrants.

## Methods

### Data

We conducted a retrospective, open-cohort study based on Swedish total population register data. Data sources were linked using pseudonymized personal identification numbers. These included the Total Population Register and Longitudinal Integrated Database for Health Insurance and Labour Market Studies for annual information on socioeconomic, social insurance and migration-related factors (including for censoring);^26^ the Prescribed Drug Register (PDR) for prescription data;^27^ and the Cause of Death Register for censoring purposes.^28^ We identified all heterosexual first-time married individuals aged 18 or older and residing in Sweden for at least 1 year between 31 December 2005 and 31 December 2016. Follow-up began on 31 December 2005, year of migration to Sweden (if already married) or year of marriage; and continued until the outcome event, end of the study period or individuals’ death or emigration, whichever came first. Individuals who remarried before or during follow-up and return migrants (i.e. foreign-born individuals that had lived in Sweden prior to the study period) were excluded. The study was approved by the Stockholm Regional Ethical Review Board (decision no. 2017/716-31/5).

### Variables

For the outcome, we measured time to first prescription of psychotropic medication, including antidepressants and anxiolytics (Anatomical Therapeutic Chemical or ATC Codes N05B and N06A, respectively), presented together (i.e. whichever came first) and separately. Native and immigrant (i.e. foreign-born) individuals were characterized by their marriage composition, as immigrant or native intramarriages (where both individuals are either immigrants or natives, respectively) and intermarriages (between one immigrant and one native individual), with gender-specific index persons. Immigrants were further characterized by their region of origin, classified as regions predominantly consisting of Organization for Economic Cooperation and Development (OECD) member states (i.e. Europe, North America and Oceania) and non-OECD-predominant regions (i.e. Africa, Asia, South America, Middle East and stateless/other) based on previous operationalizations.^29^ All index persons were examined for educational attainment (low: up to 2 years of upper secondary education; medium: up to 2 years of university college; high: graduate/postgraduate studies; and missing) and individual disposable income (quartiles calculated based on the study population’s income distribution for each calendar year) measured as time-varying covariates with 1-year lags.^25^ Finally, we included information on presence of a minor child in the household (i.e. <18 years old; dichotomous, lagged and time-varying), time in marriage (years at baseline) and incidence of divorce, partner’s emigration or partner’s death (at any point during follow-up; dichotomous) to account for familial and marital differences across marriage groups.

### Statistical analysis

We conducted a survival analysis using Cox regression models with age as the time scale. Gender-stratified models were calculated using marriage composition as the primary exposure, with additional inclusion of covariates relating to socioeconomic factors (educational attainment and individual disposable income); family structure (presence of a minor child in the household); and marriage length and quality (time in the marriage, occurrence of divorce and partner’s emigration or death). Subgroup analyses by immigrants’ regions of origin were also conducted. In a sensitivity analysis, we examined alternate categories of immigrant origin (Nordic, other European and non-European) to further investigate the role of regional differences. Finally, we excluded couples that experienced divorce or a partner’s emigration or death during the study period, to remove potential bias from negative marital experiences. Results are presented as hazard ratios (HR) and 95% confidence intervals (95% CI) with robust standard errors to account for non-proportional hazards.^30^ Analyses were conducted using Stata Version 15.1 (StataCorp, College Station, Texas).

## Results

The study population consisted of 1 867 322 first-time married couples followed up for 27 395 773 person-years (PY) (see table 1 for descriptives). These included native–native intramarriages (*n* = 1 400 702 dyads), native–immigrant intermarriages (with immigrant woman: *n* = 108 903 dyads; immigrant man: *n* = 80 817 dyads) and immigrant–immigrant intramarriages (*n* = 276 900 dyads). Among women, psychotropic prescription rates were highest for intramarried immigrants (49.99 per 1000 PY) and lowest for intermarried immigrants (48.57 per 1000 PY). Rates among men were highest for intramarried immigrants (35.30 per 1000 PY) and lowest for intramarried natives (31.41 per 1000 PY). Intramarried native women and men were on average older at baseline (50.35 years, standard deviation or SD = 0.01; 52.73 years, SD = 0.01; respectively) than others, with longer marriage duration upon study entry. Intermarried immigrants had the highest educational attainment and intramarried natives the highest income levels. Intramarried native and intermarried immigrant women were less likely to have a minor child at home at baseline than intramarried immigrants. During follow-up, immigrants were most likely to emigrate, intermarried couples most likely to divorce and intramarried natives most likely to be bereaved. Most intermarried immigrants were of OECD origin (women, 74.86%; men, 84.10%) whereas intramarried immigrants were more evenly of OECD and non-OECD origin. The majority of intramarried immigrants were of the same origin as their partner.

**Table 1:** Distribution of time-at-risk for first-time marriages of individuals residing in Sweden 31 December 2005–31 December 2016, by gender of index person and marital composition (*n* = 1 867 322 couples; 27 395 773 person-years)

		Native intramarriage	Intermarriage	Immigrant intramarriage
Index person (woman)		Native women	Immigrant women	Immigrant women
Sample size (*n*)		1 400 702	108 903	276 900
Time-at-risk (in 1000 PY)		10 316.95	744.21	1541.22
Prescriptions (rates per 1000 PY)	Either	49.68	48.57	49.99
	Antidepressants	34.68	33.03	34.21
	Anxiolytics	29.21	29.78	31.15
Age at baseline (mean years, SD)		50.35 (0.01)	45.09 (0.05)	38.67 (0.03)
Time in marriage (mean years, SD)		21.52 (0.02)	14.49 (0.05)	13.86 (0.03)
Educational attainment	Low	18.32	17.73	24.15
	Medium	46.95	38.66	31.96
	High	34.63	39.00	24.57
	Missing	0.10	4.61	19.32
Individual disposable income	Quartile 1	25.52	41.69	62.16
	Quartile 2	30.37	24.70	20.84
	Quartile 3	27.79	20.99	12.39
	Quartile 4	16.32	12.62	4.61
Minor child in household		39.85	41.03	53.24
Partner emigrated		0.08	0.19	1.93
Divorced		5.56	9.35	9.69
Bereaved		10.48	8.92	5.34
Region of origin	OECD	–	74.86	53.89
	Non-OECD	–	25.14	46.11
**Index person (man)**		**Native men**	**Immigrant men**	**Immigrant men**
Sample size (*n*)		1 400 702	80 817	276 900
Time-at-risk (in 1000 PY)		11 205.16	577.23	1660.24
Prescriptions (rates per 1000 PY)	Either	31.41	32.15	35.30
	Antidepressants	20.66	21.30	23.08
	Anxiolytics	19.65	20.07	22.03
Age at baseline (mean years, SD)		52.73 (0.01)	46.27 (0.06)	42.77 (0.03)
Time in marriage (mean years, SD)		21.52 (0.02)	14.56 (0.06)	13.78 (0.03)
Educational attainment	Low	23.13	17.59	22.99
	Medium	50.74	45.56	36.35
	High	25.98	31.52	25.24
	Missing	0.16	5.33	15.42
Individual disposable income	Quartile 1	8.60	23.23	43.99
	Quartile 2	17.41	18.60	21.31
	Quartile 3	28.34	24.93	20.26
	Quartile 4	45.64	33.24	14.44
Minor child in household		40.32	43.87	48.91
Partner emigrated		0.03	0.07	1.73
Divorced		5.84	10.98	9.56
Bereaved		5.04	3.62	2.12
Region of origin	OECD	–	84.10	54.21
	Non-OECD	–	15.90	45.79
	Same as partner	–	–	94.95

OECD, Organization for Economic Cooperation and Development; PY, person-years; SD, standard deviation. Values presented as distribution of time-at-risk (%) for either prescription, unless otherwise specified. Age, time in marriage, educational attainment, individual disposable income and minor child in household variables presented at baseline. All other variables presented for full study period.

**Figure 1:**
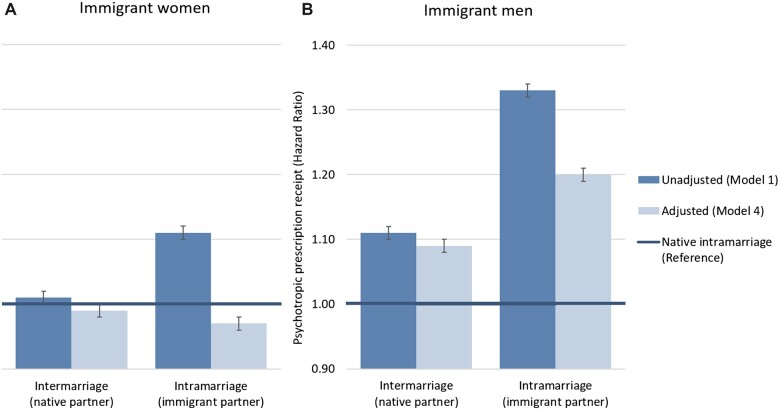
Risk of antidepressant or anxiolytic prescription receipt for (A) immigrant women and (B) immigrant men, by marital composition, relative to intramarried natives. Presented as hazard ratios with 95% confidence intervals. Model 1: unadjusted. Model 4: adjusted for socioeconomic factors, presence of a minor child in the household and marital experiences (time in marriage, partner’s emigration, divorce and bereavement). For all point estimates and confidence intervals for Models 1–4, see Supplementary table S1

For women, unadjusted models revealed no difference in psychotropic prescription hazards in intermarried immigrants, but higher hazards in intramarried immigrants (HR 1.11, 95% CI 1.10–1.12), compared to intramarried natives (figure 1A, Supplementary table S1, Model 1). After controlling for all covariates, hazards for inter- and intramarried immigrants became equal or lower than those of intramarried natives (figure 1A, Supplementary table S1, Models 2–4). Socioeconomic factors attenuated findings for intramarried immigrants in particular. Among men, intramarried immigrants (HR 1.33, 95% CI 1.32–1.34), then intermarried immigrants (HR 1.11, 95% CI 1.10–1.13), had higher prescription hazards than intramarried natives (figure 1B, Supplementary table S1, Model 1). After controlling for socioeconomic factors, intramarried immigrant men’s hazards were partly attenuated but remained higher than for intermarried immigrants and references, with little change after adjustment for familial and marital factors (figure 1B, Supplementary table S1, Models 2–4).

Subgroup analyses by region of origin revealed that immigrant women’s own origins were most predictive of psychotropic prescription levels, with equal or lower hazards among non-OECD-origin women, and equal or greater hazards in OECD-origin women, relative to natives (table 2, Models 1–4). Within each region-of-origin group, immigrant women’s intermarriage with natives or immigrants of another origin corresponded to intermediate hazards between native references and intramarried immigrants. Among men, immigrants of non-OECD origin had the greatest prescription hazards, increasing continuously by the similarity of their partners’ origins (e.g. native partner: HR 1.28, 95% CI 1.24–1.33; OECD-origin partner: HR 1.33, 95% CI 1.26–1.40; and non-OECD-origin partner: HR 1.46, 95% CI 1.44–1.48). Similarly, OECD-origin men with OECD partners had higher hazards than their intermarried peers, all with some attenuation after adjustment.

**Table 2:** Risk of antidepressant or anxiolytic prescription receipt, by gender of index person and marital composition (nativity/region of origin)

	Immigrant women				Immigrant men			
	Model 1	Model 2	Model 3	Model 4	Model 1	Model 2	Model 3	Model 4
	HR (95% CI)	HR (95% CI)	HR (95% CI)	HR (95% CI)	HR (95% CI)	HR (95% CI)	HR (95% CI)	HR (95% CI)
Marital composition (ref. native intramarriage)								
OECD immigrants								
Intermarriage (native partner)	1.08 (1.07–1.10)	1.07 (1.06–1.08)	1.07 (1.06–1.08)	1.08 (1.07–1.10)	1.09 (1.07–1.11)	1.07 (1.06–1.09)	1.07 (1.06–1.09)	1.08 (1.06–1.09)
Intermarriage (immigrant partner, non-OECD)	0.96 (0.92–1.01)	0.91 (0.87–0.95)	0.91 (0.87–0.95)	0.87 (0.83–0.91)	1.01 (0.95–1.08)	0.94 (0.88–1.00)	0.94 (0.88–1.00)	0.96 (0.90–1.03)
Intramarriage (immigrant partner, OECD)	1.18 (1.17–1.19)	1.08 (1.07–1.10)	1.08 (1.07–1.10)	1.05 (1.04–1.06)	1.26 (1.25–1.28)	1.18 (1.17–1.20)	1.18 (1.17–1.20)	1.16 (1.14–1.17)
Non-OECD immigrants								
Intermarriage (native partner)	0.76 (0.74–0.78)	0.70 (0.68–0.72)	0.70 (0.68–0.71)	0.70 (0.68–0.72)	1.28 (1.24–1.33)	1.23 (1.18–1.27)	1.23 (1.18–1.27)	1.16 (1.12–1.21)
Intermarriage (immigrant partner, OECD)	0.75 (0.71–0.80)	0.68 (0.64–0.72)	0.68 (0.64–0.72)	0.67 (0.63–0.71)	1.33 (1.26–1.40)	1.23 (1.17–1.30)	1.24 (1.17–1.30)	1.18 (1.12–1.25)
Intramarriage (immigrant partner, non-OECD)	1.04 (1.03–1.05)	0.93 (0.91–0.94)	0.93 (0.91–0.94)	0.87 (0.86–0.89)	1.46 (1.44–1.48)	1.31 (1.29–1.33)	1.32 (1.30–1.33)	1.28 (1.26–1.30)

95% CI, 95% confidence interval; HR, hazard ratio; OECD, Organization for Economic Cooperation and Development. Controls: Model 1: marital composition; Model 2: marital composition and socioeconomic factors (educational attainment and disposable income); Model 3: marital composition, socioeconomic factors and presence of a minor child in the household; Model 4: marital composition, socioeconomic factors, presence of a minor child in the household and marital experiences (time in marriage, partner’s emigration, divorce and bereavement). Note: HRs for covariates not shown.

In further subgroup analyses by prescription type, antidepressant prescription hazards were lower in inter- and intramarried immigrant women than intramarried natives, even before adjusting for covariates (Supplementary table S2A). Inter- and especially intramarried immigrant men had greater antidepressant hazards than natives, partly attenuated after controlling for all covariates. For anxiolytic prescriptions, both inter- and intramarried immigrant women had greater hazards than natives, whereas men exhibited the same patterns as in the main analysis (Supplementary table S2B).

Sensitivity analyses on alternate regional groupings revealed further heterogeneity in prescription hazards in intermarried individuals (Supplementary table S3). As with the OECD-based categories, intermarried women from Nordic and other European countries showed higher prescription hazards, and women of non-European origin lower hazards, than intramarried natives. Sensitivity analyses for men similarly reflected the findings of the main analyses, suggesting that greater differences with the native population (i.e. for Nordic, other European, then non-European immigrants) corresponded to higher prescription hazards. Additional sensitivity analyses on couples that remained intact throughout the study period (i.e. no divorce, bereavement or partner’s emigration) revealed higher hazards among those with at least one immigrant individual, relative to the main analyses (Supplementary table S4).

## Discussion

This study found varied levels of psychotropic prescriptions by native–immigrant marital composition in Sweden. Among women, all marriage categories had greater hazards than intramarried native references until adjustment for socioeconomic, familial and marital covariates. Immigrant women’s region of origin appeared to be a strong predictor of prescription hazards, with some protective effects of intermarriage with natives or immigrants of other origins, albeit with modest estimates. Meanwhile, men in immigrant intramarriages had the highest hazards of psychotropic prescriptions relative to native intramarried men, even after adjustment for covariates. For intermarried immigrant men, prescription hazards increased with the degree of similarity with their partner’s region of origin, i.e. whether they were natives, immigrants of other origins or immigrants of the same origin.

Our finding that intermarried immigrants have lower risks of psychotropic prescriptions than intramarried immigrants, but greater than intramarried natives, is supported by previous evidence on self-reported depression among intermarried and intramarried Western immigrants in Europe.^24^ This suggests that integration can act as a ‘leveler’ of health inequalities between natives and immigrants. However, our findings for non-OECD immigrants do not align with previous evidence that shows greater risks of depression^24^ and suicide^25^ in intermarriages compared to intramarriages, even after controlling for relevant covariates. This inconsistency suggests that the association between intermarriage and mental health is contingent on specific health outcomes. Future studies should disentangle whether this is due to differences in psychiatric needs and actual healthcare use, or in the underlying integration mechanisms, including through socioeconomic, social and cultural pathways.

Evidence suggests that socioeconomic ‘premiums’ associated with intermarriage are predominantly attributable to the selection of socioeconomically advantaged (and, by extension, healthier) immigrants into marriages with natives.^13^^,^^14^ Yet, intermarriage could also influence prescription patterns by promoting immigrants’ socioeconomic status (e.g. labour market attachment), and, in turn, decreasing their underlying health needs. Due to a lack of pre-marital data, we were unable to differentiate between selection-based (i.e. confounding) and marriage-related (i.e. mediating) premiums in our analyses. However, the role of socioeconomic mechanisms in intermarriage-related health differences was evident, given that these factors fully attenuated differences in inter- and intramarried immigrant women’s prescription risks. Inter- and intramarried men appeared to have prescription differences even after socioeconomic adjustments, suggesting the presence of other integration-related factors not captured in this study.

Among other explanations, socio-cultural selection and adaptation could play a role in the intermarriage-health association, especially given that intermarriage is more common among culturally similar immigrants and natives, leading to fewer obstacles for integration.^31^ On the one hand, intermarriage could reduce immigrants’ risks of psychotropic prescription receipt by improving their psychological health via acculturative processes, expanded social networks and reduced experiences of intercultural conflict, including discrimination.^18^ On the other hand, intermarriage could increase psychotropic prescription rates by encouraging healthcare-seeking through the acquisition of native norms, language and institutional knowledge, as indicated by evidence on acculturation^20^^,^^21^^,^^32^ and duration of residence,^3^^,^^5^ albeit with less conclusive evidence for intermarriage so far.^22^ Thus, our seemingly conflicting findings of greater psychotropic prescription risks in intermarried than intramarried immigrant women, but lower risks in intermarried than intramarried immigrant men, could be explained by the joint influence of socio-cultural selection and adaptation with regards to health needs (i.e. improved health) and healthcare-related behaviours (i.e. increased uptake), alongside the co-occurring trend of socioeconomic-related health gains through intermarriage.

Finally, we examined whether the influence of intermarriage on health differed by immigrants’ regions of origin. Previous evidence from the Nordic region suggests that Western (i.e. OECD-origin) immigrants use psychological and psychiatric care at similar rates as natives, whereas non-Western (i.e. non-OECD-origin) immigrants have lower uptake.^33^ This was supported by our findings for women. Yet, we found greater levels of prescription receipt in immigrant men of non-OECD than OECD origins, irrespective of their marriage composition, as well as in sensitivity analyses for intermarried non-European immigrant men, relative to intermarried Nordic and other European immigrants. Given the lack of consistent findings by region of origin, it stands to reason that the investigated health outcomes may be influenced by a combination of the aforementioned integration pathways.

### Strengths and limitations

This study used high-quality longitudinal register data to assess the link between native–immigrant marital composition and health, interpreting marriage as a representative state for the adult population and intermarriage as an apt proxy for integration in adulthood. Yet, interethnic couples (and by extension, couples of different nativities) tend to have unique relationship constellations through prolonged cohabitation and greater likelihood of divorce and remarriage.^34–36^ Accurate information on cohabitation status was lacking for the full study period, given that its primary source, the Swedish Dwelling Register, was not established until 2011, and we chose to focus on first-time marriages to avoid potential bias from prior marriages, thus likely underestimating the population of interethnic couples. Studies have also shown that these intercultural marriages are associated with worse relationship quality^36^ and long-term decreases in life satisfaction,^37^ potentially manifesting as poorer health. Although we cannot account for the bias of excluding remarried couples, controlling for familial and marital proxies, and excluding individuals with negative marital experiences in our sensitivity analyses, did not notably alter our estimates. Our results may have also been biased through selection into intermarriage by socioeconomic position.^12–14^ Since we were unable to measure pre-marital socioeconomic factors—especially for immigrants that arrived in Sweden already married—we controlled for lagged, time-varying factors during marriage, potentially accounting for both confounding and mediation in the intermarriage-health association. Similarly, due to a lack of power to conduct subgroup analyses describing country-level socio-cultural differences,^31^ we cannot draw conclusions regarding the role of these factors in the intermarriage-health association. Finally, by removing return migrants from the analyses, we may have overlooked a portion of the immigrant population with unique integration and health characteristics. Future studies should thus aim to disentangle immigrants’ pre- and post-marital experiences from their integration consequences, while also considering alternate indicators of integration in different country contexts to understand the generalizability of the results.

The use of psychotropic prescription data allowed us to examine a unique measure of mental health, although this was complicated by the interaction of underlying health needs and healthcare-seeking behaviours. Medications may have been prescribed for a range of symptoms of varying severity, but unfortunately neither data on the dosage nor the source of the prescription (e.g. general practitioner, psychiatrist) was available. Immigrants have also been found to have especially low levels of psychotropic medication compliance and adherence.^38^ While we could not determine whether the medication was in fact consumed, the PDR only includes data on dispensed medications, indicating some level of compliance. As a consequence of these data limitations, we relied on a basic estimate of time to first prescription^38^ but future investigations should consider dosages and repeat prescriptions, together with other clinical indicators, to better indicate the severity of underlying mental health problems. In addition, the analyses may have been influenced by informative censoring for those who committed suicide in lieu of seeking care, especially as these risks vary between inter- and intramarriages.^25^ However, it is beyond the scope of this study to quantify their impact on psychotropic prescription risks. Alternate indicators of mental health should be examined to elaborate on these mechanisms.

## Conclusions

Immigrants represent a diverse population of individuals, with varied mental health risks by differing levels of integration. This study aimed to explore immigrants’ psychotropic prescription receipt by a common proxy of integration, intermarriage between immigrants and natives. The study revealed intermediate levels of psychotropic prescription receipt in intermarried immigrants, especially immigrant men, relative to intramarried natives and immigrants. This suggests that integration could be protective for immigrant health, whether through socioeconomic gains, reduced intercultural conflict or greater healthcare-seeking. The findings of this study emphasize the complexity of intermarriage as a ‘litmus test’ for integration,^10^^,^^39^ calling for further investigations of its intersecting social, cultural and socioeconomic mechanisms.

## Supplementary data

Supplementary data are available at *EURPUB* online.

## Funding

This work was funded by the Swedish Research Council for Health, Working Life and Welfare (FORTE) grant 2016–07128.

*Conflicts of interest*: The authors declare that they have no conflicts of interest.

## Supplementary Material

ckac158_Supplementary_Data

## Data Availability

The data are available from Statistics Sweden under license for the current study, and are not publicly available. Immigrants’ mental health needs and healthcare uptake have been found to vary by their gender, region of origin and duration of residence in the host country, yet little is known about how these vary by immigrants’ integration status. This study examines prescription risks for psychotropic medications by native–immigrant intermarriage as a proxy for integration in Sweden. Findings reveal that intermarried immigrants have lower levels of psychotropic prescription receipt than intramarried immigrants, especially among men, albeit greater than among intramarried natives. Public health efforts to reduce native–immigrant mental health inequalities should consider the role of immigrants’ integration status.
