# Supplementary material for: Mental health by native–immigrant intermarriage in Sweden: a register-based retrospective cohort study
Source: Eur J Public Health. 2022 Nov 15;32(6):877–83. doi: 10.1093/eurpub/ckac158 (PMC9713391; doi:10.1093/eurpub/ckac158)
Supplement: ckac158_Supplementary_Data [file ckac158_supplementary_data.docx]

**Table S1. Main analysis:** Risk of antidepressant or anxiolytic prescriptions, by gender of index person and marital composition (nativity)

|  | Immigrant women | | | |
| --- | --- | --- | --- | --- |
|  | **Model 1** | **Model 2** | **Model 3** | **Model 4** |
|  | **HR (95% CI)** | **HR (95% CI)** | **HR (95% CI)** | **HR (95% CI)** |
| **Marital composition**  (ref. native intramarriage) |  | | | |
| Intermarriage  (native partner) | 1.01 (1.00-1.02) | 0.98 (0.97-0.99) | 0.98 (0.97-0.99) | 0.99 (0.98-1.01) |
| Intramarriage  (immigrant partner) | 1.11 (1.10-1.12) | 1.01 (1.01-1.02) | 1.01 (1.01-1.02) | 0.97 (0.96-0.98) |
|  | **Immigrant men** | | | |
|  | **Model 1** | **Model 2** | **Model 3** | **Model 4** |
|  | **HR (95% CI)** | **HR (95% CI)** | **HR (95% CI)** | **HR (95% CI)** |
| **Marital composition**  (ref. native intramarriage) |  | | | |
| Intermarriage  (native partner) | 1.11 (1.10-1.13) | 1.09 (1.08-1.11) | 1.09 (1.08-1.11) | 1.09 (1.07-1.10) |
| Intramarriage  (immigrant partner) | 1.33 (1.32-1.34) | 1.23 (1.21-1.24) | 1.23 (1.22-1.24) | 1.20 (1.19-1.21) |

Abbreviations: CI: Confidence Interval; HR: Hazard Ratio. Controls: Model 1: Marital composition; Model 2: Marital composition and socioeconomic factors (educational attainment, disposable income); Model 3: Marital composition, socioeconomic factors and presence of a minor child in the household; Model 4: Marital composition, socioeconomic factors, presence of a minor child in the household and marital experiences (time in marriage, partner’s emigration, divorce, bereavement). Note: HRs for covariates not shown.

**Table S2. Sub-group analyses:** Risk of antidepressant and anxiolytic prescriptions, by gender and marital composition

| A. Antidepressants | Immigrant women | | Immigrant men | |
| --- | --- | --- | --- | --- |
|  | **Model 1** | **Model 2** | **Model 1** | **Model 2** |
|  | **HR (95% CI)** | **HR (95% CI)** | **HR (95% CI)** | **HR (95% CI)** |
| **Marital composition**  (ref. native intramarriage) |  | | | |
| Intermarriage  (native partner) | 0.96 (0.94-0.97) | 0.94 (0.93-0.95) | 1.10 (1.08-1.12) | 1.06 (1.04-1.08) |
| Intramarriage  (immigrant partner) | 1.02 (1.01-1.03) | 0.88 (0.87-0.89) | 1.26 (1.24-1.27) | 1.11 (1.10-1.13) |
| **B. Anxiolytics** | **Immigrant women** | | **Immigrant men** | |
|  | **Model 1** | **Model 2** | **Model 1** | **Model 2** |
|  | **HR (95% CI)** | **HR (95% CI)** | **HR (95% CI)** | **HR (95% CI)** |
| **Marital composition**  (ref. native intramarriage) |  | | | |
| Intermarriage  (native partner) | 1.08 (1.07-1.10) | 1.05 (1.04-1.07) | 1.14 (1.12-1.16) | 1.11 (1.09-1.13) |
| Intramarriage  (immigrant partner) | 1.26 (1.25-1.27) | 1.15 (1.13-1.16) | 1.40 (1.39-1.42) | 1.29 (1.27-1.30) |

Abbreviations: CI: Confidence Interval; HR: Hazard Ratio. Model 1: Marital composition; Model 4: Marital composition, socioeconomic factors, presence of a minor child in the household, marital experiences (time in marriage, partner’s emigration, divorce, bereavement). Note: HRs for covariates not shown.

**Table S3. Sensitivity analysis:** Risk of either antidepressant or anxiolytic prescriptions, by gender and marital composition (for native-immigrant intermarriages, by regions of origin)

|  | Immigrant women | | Immigrant men | |
| --- | --- | --- | --- | --- |
|  | **Model 1** | **Model 2** | **Model 1** | **Model 2** |
|  | **HR (95% CI)** | **HR (95% CI)** | **HR (95% CI)** | **HR (95% CI)** |
| **Marital composition**  (ref. native intramarriage) |  | | | |
| Intermarriage (Nordic immigrant, native partner) | 1.04 (1.03-1.06) | 1.05 (1.03-1.07) | 1.03 (1.00-1.06) | 1.02 (1.00-1.05) |
| Intermarriage (Other European immigrant, native partner) | 1.13 (1.11-1.15) | 1.12 (1.10-1.14) | 1.13 (1.11-1.16) | 1.11 (1.09-1.14) |
| Intermarriage (Non-European immigrant, native partner) | 0.80 (0.78-0.82) | 0.75 (0.73-0.77) | 1.24 (1.20-1.28) | 1.14 (1.11-1.18) |
| Intramarriage (all immigrants) | 1.11 (1.10-1.12) | 0.97 (0.96-0.98) | 1.33 (1.32-1.34) | 1.20 (1.19-1.21) |

Abbreviations: CI: Confidence Interval; HR: Hazard Ratio. Model 1: Marital composition; Model 4: Marital composition, socioeconomic factors, presence of a minor child in the household, and marital experiences (time in marriage, partner’s emigration, divorce, bereavement). Note: HRs for covariates not shown.

**Table S4. Sensitivity analysis:** Risk of either antidepressant or anxiolytic prescriptions, by gender and marital composition, for intact couples (i.e., no emigration, death or divorce)

|  | Immigrant women | | Immigrant men | |
| --- | --- | --- | --- | --- |
|  | **Model 1** | **Model 2** | **Model 1** | **Model 2** |
|  | **HR (95% CI)** | **HR (95% CI)** | **HR (95% CI)** | **HR (95% CI)** |
| **Marital composition**  (ref. native intramarriage) |  | | | |
| Intermarriage  (native partner) | 1.02 (1.01-1.04) | 1.02 (1.00-1.03) | 1.14 (1.12-1.16) | 1.13 (1.10-1.15) |
| Intramarriage  (immigrant partner) | 1.22 (1.21-1.23) | 1.06 (1.05-1.07) | 1.50 (1.48-1.52) | 1.34 (1.33-1.36) |

Abbreviations: CI: Confidence Interval; HR: Hazard Ratio. Model 1: Marital composition; Model 4: Marital composition, socioeconomic factors, presence of a minor child in the household, and marital experiences (time in marriage). Note: HRs for covariates not shown.
